# Network Meta-Analysis on the Effects of SGLT2 Inhibitors Versus Finerenone on Cardiorenal Outcomes in Patients With Type 2 Diabetes and Chronic Kidney Disease

**DOI:** 10.3389/fphar.2021.751496

**Published:** 2022-01-24

**Authors:** Li-Min Zhao, Ze-Lin Zhan, Jie Ning, Mei Qiu

**Affiliations:** ^1^ Department of Endocrinology, Shenzhen Longhua District Central Hospital, Shenzhen, China; ^2^ Clinical Medicine, The Second Clinical Medical College, Southern Medical University, Guangzhou, China; ^3^ Department of General Medicine, Shenzhen Longhua District Central Hospital, Shenzhen, China

**Keywords:** SGLT2 inhibitors, finerenone, type 2 diabetes, chronic kidney disease, renal failure, heart failure

## Introduction

A large randomized placebo-controlled trial reported in two articles (Bakris et al., 2020; Filippatos et al., 2021) has demonstrated that finerenone, a nonsteroidal and selective mineralocorticoid receptor antagonist, can significantly reduce the occurrences of both a composite renal outcome (defined as a composite of a sustained decrease of at least 40% in the estimated glomerular filtration rate [eGFR] from the baseline, kidney failure, or death from renal causes) and a composite cardiovascular outcome (defined as a composite of nonfatal myocardial infarction [MI], nonfatal stroke, death from cardiovascular causes, or hospitalization for heart failure [HHF]) in patients with type 2 diabetes (T2D) and chronic kidney disease (CKD), regardless of patients with or without established cardiovascular disease. Therefore, finerenone becomes a new frontier in renin-angiotensin-aldosterone system (RAAS) inhibitions for treating diabetic kidney disease (Sridhar et al., 2021).

On the other hand, two meta-analyses (Arnott et al., 2020; Qiu et al., 2020) based on placebo-controlled cardiorenal outcome trials have revealed that sodium glucose co-transporter 2 (SGLT2) inhibitors, a novel class of hypoglycemic agents, significantly reduced various cardiovascular and renal outcomes in patients with T2D, regardless of patients with or without cardiorenal diseases. Therefore, this drug class has been recommended in T2D patients with established cardiorenal disease (Buse et al., 2020) including T2D patients with CKD to prevent cardiorenal events.

Finerenone exerts its renal protective effects in patients with T2D and CKD mainly by reducing inflammation, fibrosis, and albuminuria (Chaudhuri et al., 2021; Wang et al., 2021), while the renoprotective mechanisms of SGLT2 inhibitors also include these (Chaudhuri et al., 2021; Wang et al., 2021). It is essential for us to know the relative cardiorenal efficacy between SGLT2 inhibitors and finerenone when we need to make a choice between them in clinical practice. This point appears to be particularly important in the absence of evidence regarding whether their combination therapy could lead to greater cardiorenal benefits than monotherapy in patients with T2D and CKD. Due to the lack of head-to-head trials comparing SGLT2 inhibitors with finerenone in cardiorenal endpoints, performing network meta-analysis based on indirect comparisons is an effective way to derive the estimators for the relative cardiorenal efficacy between finerenone and SGLT2 inhibitors. Therefore, we conducted this network meta-analysis based on placebo-controlled cardiorenal outcome trials of SGLT2 inhibitors and finerenone, aiming to assess the relative efficacy of SGLT2 inhibitors versus finerenone on cardiorenal endpoints in patients with T2D and CKD.

## Methods

We conducted this network meta-analysis in accordance with the Preferred Reporting Items for Systematic Reviews and Meta-Analyses (PRISMA) extension statement for network meta-analyses (Hutton et al., 2015). The studies we included in this network meta-analysis were large cardiovascular or renal outcome trials which compared any SGLT2 inhibitor or finerenone with placebo in patients with T2D and CKD. Moreover, if relevant trials enrolling patients with T2D or CKD provided the outcome data of the subgroup of patients with T2D and CKD, these subgroup data would also be included in this meta-analysis. CKD was defined as eGFR <60 ml min^−1^–1.73 m^−2^ and/or urine albumin-creatinine ratio >300 mg/g (Wanner et al., 2018). Seven outcomes of interest were kidney function progression (KFP), HHF, major adverse cardiovascular events (MACE), nonfatal MI, nonfatal stroke, cardiovascular death (CVD), and all-cause death (ACD). KFP was defined as a composite of a sustained decrease of at least 40% in the eGFR from the baseline or a doubling of the serum creatinine level, kidney failure (a composite of end-stage kidney disease or sustained decrease in eGFR to <15 ml/min/1.73 m^2^), or renal death. If this composite outcome was not available, we used another composite renal outcome similar to this one instead. MACE was defined as a composite of CVD, nonfatal MI, or nonfatal stroke. If nonfatal MI and stroke were not available, we used total MI and stroke instead.

We searched Embase, PubMed, and Cochrane Central Register of Controlled Trials (CENTRAL) to identify related articles published before March 26th, 2021. The search terms that we mainly used were “Type 2 diabetes,” “Chronic kidney disease,” “Diabetic kidney disease,” “Diabetic nephropathy,” “SGLT2 inhibitors,” “Gliflozins,” “Sotagliflozin,” “Empagliflozin,” “Canagliflozin,” “Ertugliflozin,” “Dapagliflozin,” “Finerenone,” “Cardiovascular outcome*,” “Renal outcome*,” and “Randomized controlled trial.” Two authors independently performed study selection, data extraction, and risk of bias assessment. Risk of bias assessment was performed according to the Cochrane risk of bias assessment tool (Higgins et al., 2011). When they encountered the inconsistencies, they asked for the arbitration of a third author.

From included articles, we extracted the aggregated two-category data (i.e., the numbers of subjects developing events of interest and those of total subjects in the intervention group and in the placebo group) to conduct conventional meta-analysis and network meta-analysis, respectively. Treatment effects were presented as risk ratio (RR) and 95% confidence interval (CI). Conventional meta-analysis was done using the fixed-effects inverse variance method and the random-effects DerSimonian and Laird method (DerSimonian and Kacker, 2007), respectively. I^2^ statistic was calculated to measure statistical heterogeneity. If I^2^ was more than 50%, we would report random-effects results. Otherwise, we would report fixed-effects results. Network meta-analysis was done using the restricted maximum likelihood method within the frequentist framework. We completed all the data analyses in Stata/MP (version 16.0), with implementation of conventional meta-analysis via the *metan* command and implementation of network meta-analysis via a series of *network* commands.

## Results

We finally included 14 articles (Zinman et al., 2015; Wanner et al., 2016; Neal et al., 2017; Wanner et al., 2018; Neuen et al., 2018; Perkovic et al., 2019; Mahaffey et al., 2019; Wiviott et al., 2019; Bakris et al., 2020; Cannon et al., 2020; Filippatos et al., 2021; Wheeler et al., 2021; Cherney et al., 2021; Bhatt et al., 2021) reporting a total of 8 large placebo-controlled randomized trials, and from these included articles (Zinman et al., 2015; Wanner et al., 2016; Neal et al., 2017; Neuen et al., 2018; Wanner et al., 2018; Perkovic et al., 2019; Mahaffey et al., 2019; Wiviott et al., 2019; Bakris et al., 2020; Cannon et al., 2020; Filippatos et al., 2021; Wheeler et al., 2021; Cherney et al., 2021; Bhatt et al., 2021), we extracted the relevant original data used for meta-analysis, which are presented in Supplementary Table S1. Included eight trials were all with low risk of bias (Supplementary Figure S1) and consisted of one trial (Zinman et al., 2015; Bakris et al., 2020; Filippatos et al., 2021) of finerenone (i.e., FIDELIO-DKD) and seven trials (Wanner et al., 2016; Neal et al., 2017; Neuen et al., 2018; Wanner et al., 2018; Mahaffey et al., 2019; Perkovic et al., 2019; Wiviott et al., 2019; Cannon et al., 2020; Bhatt et al., 2021; Cherney et al., 2021; Wheeler et al., 2021) of gliflozins, which were EMPA-REG OUTCOME (Zinman et al., 2015; Wanner et al., 2016; Wanner et al., 2018), CANVAS Program (Neal et al., 2017; Neuen et al., 2018), CREDENCE (Mahaffey et al., 2019; Perkovic et al., 2019), DECLARE–TIMI 58 (Wiviott et al., 2019), DAPA-CKD (Cannon et al., 2020), VERTIS CV (Wheeler et al., 2021; Cherney et al., 2021), and SCORED (Bhatt et al., 2021). These seven gliflozin trials (Zinman et al., 2015; Wanner et al., 2016; Neal et al., 2017; Neuen et al., 2018; Wanner et al., 2018; Mahaffey et al., 2019; Perkovic et al., 2019; Wiviott et al., 2019; Cannon et al., 2020; Bhatt et al., 2021; Cherney et al., 2021; Wheeler et al., 2021) involved a total of 13,246 patients with T2D and CKD receiving gliflozins versus 11,741 receiving placebo, while the FIDELIO-DKD trial (Bakris et al., 2020; Filippatos et al., 2021) involved a total of 2,833 patients with T2D and CKD receiving finerenone versus 2,841 receiving placebo. MACE was defined consistently across included trials, whereas the definitions of KFP were slightly different across included trials but were similar enough to be used in meta-analysis. Those definitions of KFP among included trials are detailed in Supplementary Table S2.

**FIGURE 1:**
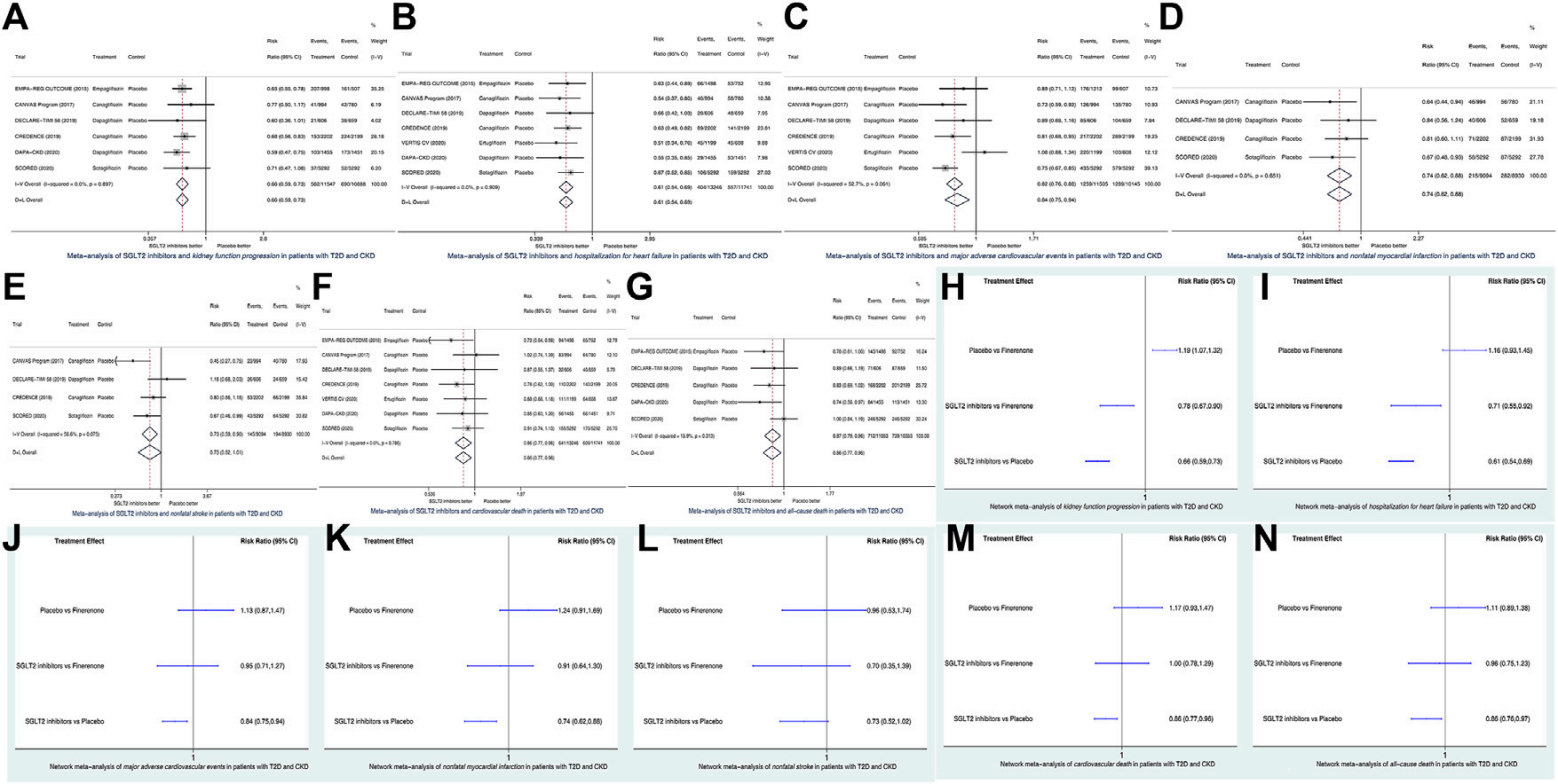
Conventional meta-analysis **(A–G)** and network meta-analysis **(H–N)** of seven cardiorenal outcomes of interest. SGLT2, sodium glucose co-transporter 2; CI. confidence interval; T2D, type 2 diabetes; CKD, chronic kidney disease.

Conventional meta-analysis results showed that in patients with T2D and CKD, compared to placebo, SGLT2 inhibitors significantly reduced the risks of KFP (HR 0.66, 95% CI 0.59–0.73, according to fixed-effects model due to I^2^ = 0%; Figure 1A), HHF (HR 0.61, 95% CI 0.54–0.69, according to the fixed-effects model due to I^2^ = 0%; Figure 1B), MACE (HR 0.84, 95% CI 0.75–0.94, according to the random-effects model due to I^2^ = 52.7%; Figure 1C), nonfatal MI (HR 0.74, 95% CI 0.62–0.88, according to the fixed-effects model due to I^2^ = 0%; Figure 1D), CVD (HR 0.86, 95% CI 0.77–0.96, according to the fixed-effects model due to I^2^ = 0%; Figure 1F), and ACD (HR 0.87, 95% CI 0.79–0.96, according to fixed-effects model due to I^2^ = 15.9%; Figure 1G) but did not significantly affect the risk of nonfatal stroke (HR 0.73, 95% CI 0.52–1.01, according to the random-effects model due to I^2^ = 56.6%; Figure 1E).

Network meta-analysis results showed that in patients with T2D and CKD, compared to finerenone, SGLT2 inhibitors significantly reduced the risks of KFP (HR 0.78, 95% CI 0.67–0.90; Figure 1H) and HHF (HR 0.71, 95% CI 0.55–0.92; Figure 1I), whereas SGLT2 inhibitors versus finerenone had the similar risks of MACE (HR 0.95, 95% CI 0.71–1.27; Figure 1J), nonfatal MI (HR 0.91, 95% CI 0.64–1.30; Figure 1K), nonfatal stroke (HR 0.70, 95% CI 0.35–1.39; Figure 1L), CVD (HR 1.00, 95% CI 0.78–1.29; Figure 1M), and ACD (HR 0.96, 95% CI 0.75–1.23; Figure 1N). As revealed by the FIDELIO-DKD trial (Bakris et al., 2020; Filippatos et al., 2021), finerenone versus placebo significantly reduced the risk of KFP (Figure 1H) but did not significantly affect the risks of the other six outcomes of interest (Figures 1J–N).

As is shown in Supplementary Figure S2, all the network plots for the seven outcomes assessed in this network meta-analysis did not have any closed loop. This suggested that there was only indirect evidence but not direct evidence between finerenone and SGLT2 inhibitors. Thus, there was no need to do an inconsistency test for this network meta-analysis.

## Discussion

In the absence of direct evidence between SGLT2 inhibitors and finerenone, this study, for the first time, provided the estimators of the relative efficacy of SGLT2 inhibitors versus finerenone on renal and cardiovascular outcomes in patients with concomitant T2D and CKD by network meta-analysis incorporating large trials of gliflozins versus placebo and those of finerenone versus placebo.

Network meta-analysis results in this study revealed that gliflozins led to the greater reductions in the risks of KFP (gliflozins versus finerenone: HR 0.78, 95% CI 0.67–0.90) and HHF (gliflozins versus finerenone: HR 0.71, 95% CI 0.55–0.92) than finerenone in patients with T2D and CKD. This suggests the obvious superiority of gliflozins over finerenone in preventing renal failure and heart failure events among this vulnerable population.

Although network meta-analysis results in this study revealed that gliflozins had similar risks of MACE, nonfatal MI, CVD, and ACD compared to finerenone, conventional meta-analysis results in this study revealed that gliflozins versus placebo significantly reduced the risks of these four outcomes. On the contrary, the FIDELIO-DKD trial (Bakris et al., 2020; Filippatos et al., 2021) revealed that finerenone versus placebo did not significantly affect these outcomes. These findings suggest the potential superiority of gliflozins over finerenone in preventing atherosclerotic cardiovascular and death endpoints among patients with T2D and CKD.

In the conventional meta-analyses of gliflozins, there was no heterogeneity or only mild heterogeneity found for the five outcomes of KFP, HHF, MI, CVD, and ACD, which suggests that it is a class effect that gliflozins reduce these outcomes. Oppositely, in the conventional meta-analyses of gliflozins, there was substantial heterogeneity found for the two outcomes of MACE and stroke. This may suggest that some gliflozins could reduce MACE and stroke in patients with T2D and CKD, whereas others could not. Similarly, previous studies have already revealed that the effects of gliflozins on MACE and stroke are drug-specific effects (Giugliano and Esposito, 2019; Zhao et al., 2021a). Therefore, future studies comparing different gliflozins and finerenone in MACE and stroke would be clinically meaningful.

In this study, gliflozins were observed with greater reductions of cardiorenal events, especially renal and cardiac failure events, than finerenone, for which the possible reason is as follows: finerenone produces cardiorenal protection mainly by its anti-inflammatory and antifibrotic effects and the function of reducing albuminuria (Chaudhuri et al., 2021; Wang et al., 2021), whereas the mechanisms of cardiorenal protection of gliflozins, apart from the above mechanisms of finerenone, also include reducing blood glucose, blood pressure, uric acid, and oxidative stress, losing weight, having a natriuretic effect, and improving renal hyperfiltration and hypoxia (Ren and Zhang, 2018; Chaudhuri et al., 2021; Wang et al., 2021).

Three previous meta-analyses (Camilli et al., 2021; Yan et al., 2021; Zhao et al., 2021b) demonstrated that the combination therapy of SGLT2 inhibitors and RAAS inhibitors [such as angiotensin receptor neprilysin inhibitors (Camilli et al., 2021; Yan et al., 2021) and angiotensin-converting enzyme inhibitors/angiotensin receptor blockers (Zhao et al., 2021b)] led to greater cardiorenal benefits than SGLT2 inhibitor monotherapy in patients with heart failure or T2D. Finerenone is a nonsteroidal and selective mineralocorticoid receptor antagonist which belongs to RAAS inhibitors. Moreover, the combination therapy of empagliflozin and finerenone showed greater cardiovascular benefits than the empagliflozin or finerenone monotherapy in rats with preclinical hypertension-induced cardiorenal disease (Kolkhof et al., 2021). Therefore, a gliflozin combined with finerenone is promising to yield greater cardiorenal benefits in patients with diabetic kidney disease.

This study has several limitations. First, we did network meta-analyses based on indirect comparisons, and therefore, our findings require to be validated by head-to-head trials comparing gliflozins with finerenone. Second, gliflozins involved much more subjects than finerenone. This imbalance in patient numbers limited the statistical power of this network meta-analysis. Accordingly, an updated meta-analysis including more subjects treated with finerenone is needed. Third, although we extracted the outcome data of patients with T2D and CKD from gliflozins and finerenone’s trials, the cardiorenal risk of participants was possibly different among included trials. There is a need for further analysis adjusting relevant cardiorenal risk factors. Last, this study focused on cardiorenal efficacy outcomes and did not assess safety outcomes associated with gliflozins or finerenone. Thus, future studies assessing the safety of these two drug classes are meaningful.

Given the above limitations of this study, its findings may suggest that among patients with T2D and CKD, SGLT2 inhibitors are more effective than finerenone in reducing renal and cardiovascular endpoints, especially renal and cardiac failure events. However, further validation by head-to-head trials comparing finerenone with gliflozins would be beneficial. Moreover, there is a need for further studies to assess whether finerenone combined with a gliflozin yields more cardiorenal benefits than the respective monotherapy.

## Abbreviations

ACD, all-cause death; CKD, chronic kidney disease; KFP, kidney function progression; HHF, hospitalization for heart failure; MACE, major adverse cardiovascular events; MI, myocardial infarction; CVD, cardiovascular death; RR, risk ratio; CI, confidence interval; eGFR, estimated glomerular filtration rate; RAAS, renin-angiotensin-aldosterone system; PRISMA, Preferred Reporting Items for Systematic Reviews and Meta-Analyses; CENTRAL, Cochrane Central Register of Controlled Trials; SGLT2, sodium glucose co-transporter 2; T2D, type 2 diabetes.
