# Supplementary figures and images for: Network Meta-Analysis on the Effects of SGLT2 Inhibitors Versus Finerenone on Cardiorenal Outcomes in Patients With Type 2 Diabetes and Chronic Kidney Disease
Source: Front Pharmacol. 2022 Jan 24;12:751496. doi: 10.3389/fphar.2021.751496 (PMC8819058; doi:10.3389/fphar.2021.751496)

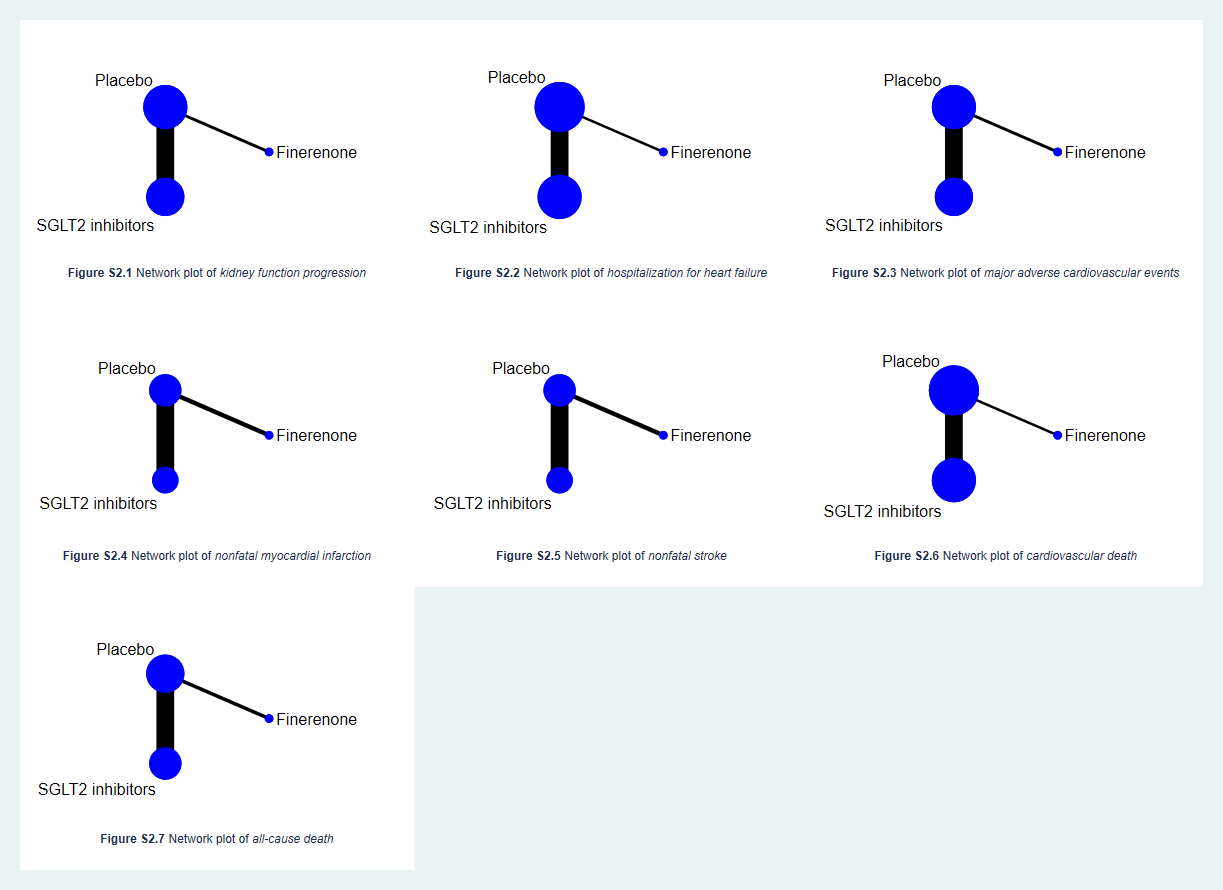

Supplement: Supplementary file 2 [file Image2.TIF]

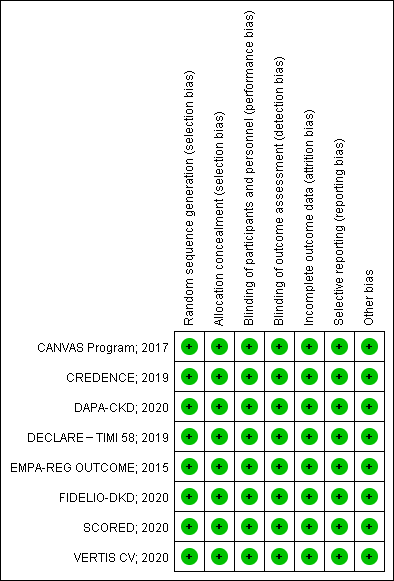

Supplement: Supplementary file 4 [file Image1.PNG]
